# What constitutes patient-centred care for women: a theoretical rapid review

**DOI:** 10.1186/s12939-019-1048-5

**Published:** 2019-11-26

**Authors:** Jessica U. Ramlakhan, Angel M. Foster, Sherry L. Grace, Courtney R. Green, Donna E. Stewart, Anna R. Gagliardi

**Affiliations:** 1 Toronto General Hospital Research Institute, University Health Network, 200 Elizabeth Street, Toronto, ON M5G 2C4 Canada; 2 Faculty of Health Sciences, University of Ottawa, 451 Smyth Road, Ottawa, ON K1H 8M5 Canada; 3 School of Kinesiology and Health Science, York University, 4700 Keele Street, Toronto, ON M3J 1P3 Canada; 4 University Health Network, 550 University Ave, Toronto, ON M5G 2A2 Canada; 5 Society of Obstetricians & Gynecologists of Canada, 2781 Lancaster Road, Suite 200, Ottawa, ON K1B 1A7 Canada; 6 Toronto General Hospital Research Institute, University Health Network, 200 Elizabeth Street, 13EN-228, Toronto, ON M5G 2C4 Canada

**Keywords:** Patient-centred care (PCC), Patient-centred care for women (PCCW), women’s health, Equality, Quality

## Abstract

**Background:**

Women experience disparities in health care delivery and outcomes. Patient-centred care for women (PCCW) is needed. This study examined how PCC has been conceptualized and operationalized in women’s health research.

**Methods:**

We conducted a theoretical rapid review of PCCW in MEDLINE, EMBASE, CINAHL and SCOPUS from 2008 to 2018 for studies involving women aged 18 years or greater with any condition, and analyzed data using an established 6-domain framework of patient-centred communication.

**Results:**

We included 39 studies, which covered the following clinical areas: maternal care, cancer, diabetes, HIV, endometriosis, dementia, distal radius fracture, overactive bladder, and lupus erythematosus. The 34 (87.2%) studies that defined or described PCC varied in the PCC elements they addressed, and none addressed all 6 PCC domains. Common domains were exchanging information (25, 73.5%) and fostering the patient-clinician relationship (22, 64.7%). Fewer studies addressed making decisions (16, 47.1%), enabling patient self-management (15, 44.1%), responding to emotions (12, 35.3%), or managing uncertainty (1, 2.9%). Compared with mixed-gender studies, those comprised largely of women more frequently prioritized exchanging information above other domains. Few studies tested strategies to support PCCW or evaluated the impact of PCCW; those that did demonstrated beneficial impact on patient knowledge, satisfaction, well-being, self-care and clinical outcomes.

**Conclusions:**

Studies varied in how they conceptualized PCCW, and in many it was defined narrowly. Few studies examined how to implement or measure PCCW; thus, we lack insight on how to operationlize PCCW. Thus, further research is needed to confirm this, and whether PCCW differs across conditions, knowledge needed to inform policies, guidelines and measures aimed at improving health care and associated outcomes for women.

## Introduction

Patient-centred care (PCC), an approach that tailors care to patient values and circumstances, has improved patient and health system outcomes for multiple conditions in a variety of settings [1–4]. PCC has been defined as care that is respectful of and responsive to individual patient preferences, needs and values, and ensures that patient values guide all clinical decisions [5]. Considerable research has conceptualized PCC. For example, a scoping review (19 studies 1994–2011) identified 25 unique PCC frameworks across which common domains pertained to patient-provider communication including information sharing, empathy, empowerment and health promotion [6]. McCormack et al. reviewed literature, observed medical encounters, interviewed patients, and engaged a 13-member expert panel to generate a PCC framework of 31 sub-domains within six interdependent domains reflecting elements of patient-provider communication: foster a healing relationship, exchange information, address patient emotions, manage uncertainty, make decisions, and enable patient self-management [7].

Despite the benefits associated with PCC, and insight on the elements of PCC and how to achieve it, many patients do not receive or experience PCC. A national survey in the United States in 2011 showed that, among 2718 responding adults aged 40 or greater with 10 common medical conditions, there was considerable variation in whether patients were involved in discussions or decision-making, key elements of PCC [8]. Sub-optimal PCC was reported by half of 1794 American cancer survivors responding in 2013 to a national survey [9]. In particular, women are less likely than men to receive PCC [10, 11]. Such disparities may be heightened by race or ethnicity in both developed [12], and less developed countries [13].

In 1995, the Fourth World Conference on Women of the United Nations revealed the need to deliver services that are sensitive to the needs and preferences of women [14], and in 2009 the World Health Organization report, “Women and Health”, emphasized the need to improve the quality of women’s health care services [15]. This remains one of 17 goals of the United Nations in the “Gender Equality in the 2030 Agenda for Sustainable Development” issued in 2018 [16].

Based on a gathering of national experts in the United States [17], and insight from women’s health experts, health system leaders, and over 200 women in Canada [18], recommendations to improve patient-centred care for women (PCCW) include developing policies, guidelines and quality measures that reflect women’s health care needs and priorities. To do so requires a thorough understanding of what constitutes PCCW. It is unclear if and how PCCW has been conceptualized because while others have reviewed PCC research, none specifically examined or reported PCC as perceived or experienced by women. Hence, the purpose of this research was to examine how PCC has been described, defined and operationalized in studies of women’s health. This may reveal important aspects of PCCW that could be addressed in policies, guidelines and quality measures aimed at improving health care and associated outcomes for women. Conversely, if research to conceptualize PCCW is lacking, then primary research is needed to explore what constitutes PCCW, as this knowledge is needed to inform PCCW planning, delivery, evaluation and improvement.

## Methods

### Approach

We conducted a theoretical review, characterized by a comprehensive search strategy, inclusion of conceptual and empirical primary sources, explicit study selection, no quality appraisal, and content analysis of included items [19]. Theoretical reviews are suitable when the aim is to describe how a given process has been conceptualized. We also adopted a rapid review approach to quickly generate a PCCW framework that could then be elaborated through primary research in a larger planned study. A rapid review is characterized by restriction to a single language (English), a short time frame (last 10 years, 2008+), exclusion of grey literature, quality of included studies is not appraised, and authors of included studies are not contacted [20, 21]. In a typical rapid review, one person performs screening and data abstraction, but we employed independent screening to enhance rigour. As there are no reporting criteria specific to theoretical reviews, we employed the Preferred Reporting Items for Systematic Reviews and Meta-Analyses criteria [22]. Data were publicly available so institutional review board approval was not needed. We did not register a protocol for this review.

### Eligibility criteria

We used a PICOT (participants, issue, comparisons, outcomes, type of publication) framework to establish eligibility criteria. Participants included at least 50.0% adult women (18+) participants, and/or clinicians (physicians, nurses) of any specialty in primary, secondary or tertiary care. The problem investigated included any specific condition not included in our previous complementary review, which addressed cardiovascular disease, mental health, and reproductive health topics prioritized by our research team at that time [23]. The current review examined other conditions to assess if PCCW differs for women facing different health care issues. The issue referred to PCC, or a synonymous term such as person-, women-, client-, or family-centred care, or approaches or strategies to promote or support PCC. PCC was viewed as compassionate, respectful care that addresses patient values and preferences, as well as information and supportive care needs, thus requiring patient-level engagement and patient-provider interaction [1–7]. Patient-centred communication is the cornerstone on which patient-centred care is built. For this reason, we focused our review on the elements of good patient-centred communication (PCC) in order to illuminate the broader application of PCCW. To do this, we adopted McCormack et al.’s conceptualization of PCC of 31 elements organized in six domains: fostering patient-clinician relationship, exchanging information, recognizing and responding to patient emotions, managing uncertainty, making decisions, and enabling patient self-management [7]. Comparisons referred to studies that explored patient or clinician views about PCCW, its barriers and how to achieve it; or studies that evaluated strategies, interventions or tools aimed at supporting PCCW.. Thus, publication type including qualitative (interviews, focus groups, qualitative case studies) and quantitative (questionnaires, randomized controlled trials, time series, before/after studies, prospective or retrospective cohort studies, case control studies) research designs, or mixed methods studies published in English language. Outcomes included but were not limited to awareness, understanding, experiences or impacts of PCCW, or determinants or factors influencing any of these functions, or the impact of strategies implemented to support or improve PCCW. Although systematic reviews were not eligible (to avoid duplication of studies included in reviews and by our search), we screened their references to identify additional eligible primary studies.

### Planning

On February 9, 2018, ARG (principal investigator) conducted a preliminary search of MEDLINE using [patient-centered care”] AND [wom#n or female] from 2008 to that date. This initial search was carried out to capture relevant studies that did not necessarily employ the term PCC, become familiar with the literature, and develop a more comprehensive search strategy. This search returned nearly 31,000 results of which many were not relevant to PCC, requiring considerable time and effort to screen. For example, many studies arbitrarily used the term PCC referring to clinical care without defining, describing or measuring PCC. Thus, we opted for a more focused search strategy to retrieve articles that specifically employed the term PCC or similar alternatives.

### Searching

Our search strategy was developed with a medical librarian and complied with the Peer Review of Electronic Search Strategy reporting guidelines (Additional file 1: Table S1) [24]. On February 26, 2018, we searched MEDLINE, EMBASE, CINAHL, and SCOPUS from 2008 to that date. We searched for studies that explicitly used the term “patient-centered”, or an alternative spelling or synonymous option. We supplemented that keyword search with Medical Subject Headings reflecting the concept of PCC to identify studies that employed a synonymous term for PCC that we had not considered, and combined those searches with terms for women. As part of our larger study, we employed the same search strategy, and separately screened for and reviewed studies of PCCW for cardiac rehabilitation, depression, and family planning, topics prioritized by our collaborators (who included health services researchers, clinician investigators, and representatives of professional societies, disease-specific foundations, quality improvement and monitoring agencies, patient advocacy groups, patients and consumers). Thus, our search strategy reflects studies of PCCW for any conditions other than those.

### Screening

To pilot test the screening process, KB (research assistant), JUR (graduate student) and ARG independently screened the first 50 titles and abstracts, then compared and discussed discrepancies to achieve a common understanding of how to apply eligibility criteria. KB and JUR independently screened titles and abstracts against eligibility criteria, and ARG resolved queries and discrepancies. Exclusion criteria were generated concurrent with screening. Studies were not eligible if participants were: less than 50% women, solely family members, caregivers, or care partners as their views do not always match those of patients, and allied health care professionals (i.e. pharmacists, dentists) or medical trainees. Studies were also excluded if based in a long term care or residential setting, where what constitutes PCCW might differ from primary, secondary or tertiary care. Studies were excluded if they concluded that PCC was necessary, or arbitrarily used PCC to refer to patient treatment interventions or management models, the illness experience rather than the care experience, or patient-reported outcomes.,. Studies solely referring to one aspect of PCC such as information needs or empathy, rather than examining PCC as a multi-dimensional construct or approach, were also excluded.

### Data extraction

JUR and ARG pilot-tested the data extraction process on 3 studies, and compared and discussed discrepancies to achieve a common understanding of what data to extract and how. JUR extracted and tabulated data on study characteristics including author, publication year, country, study objective, research design, participants, term used to refer to PCC, definition or description of PCC, and relevant findings including details of interventions implemented to promote or support PCC, and outcomes of PCC.

### Data analysis

We used summary statistics to report the number of studies published per year, and by condition, country, study design, and term used for PCC. We compared definitions or descriptions of PCC that were articulated by participants across studies and conditions, and then mapped those PCC constructs against McCormack’s PCC framework comprised of 31 elements organized in 6 domains. While it reflects the views of cancer patients including both men and women, it was chosen because it was rigorously-developed and more comprehensive than other PCC frameworks [7]. This served to compare expressed views about what constitutes PCCW specific to women against PCC constructs considered ideal by other patients and clinicians, and potentially identify PCC constructs unique to women with conditions other than cancer. Without doing so, we would not have been able to identify gaps in the way PCCW has been studied, and instead would have compiled PCC components, work already done by McCormack and others [6, 7]. To identify gaps in the way PCCW was studied, we summarized the number of domains addressed in each included study. We also compared PCC domains addressed in studies comprised largely of women and mixed-gender studies. Instruments used to measure PCC were specified. We described the impact and determinants of PCC narratively, and the number of studies that evaluated interventions designed to promote or support PCC, and details about those interventions. Research team members, which included health services researchers, physicians of various specialties and experts in women’s health, independently reviewed data and the draft manuscript, and provided feedback that shaped the interpretation of results and conclusions.

## Results

### Search results

**Fig. 1:**
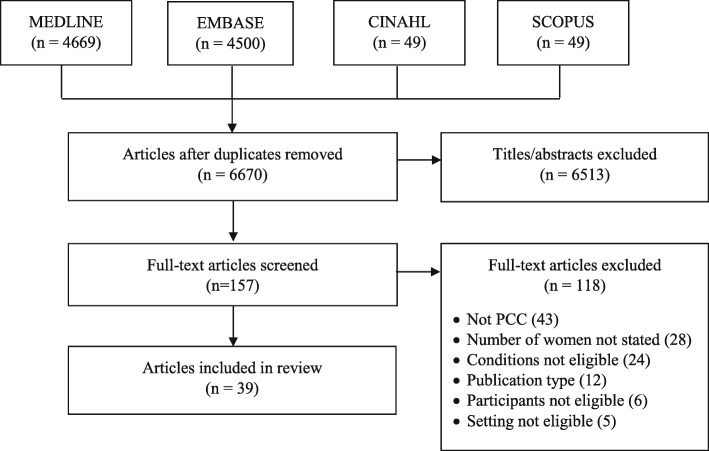
PRISMA diagram

We identified a total of 9267 studies, from which 6670 unique studies remained after removal of duplicates. Screening of titles and abstracts eliminated 6513 studies. Screening of 157 full-text articles eliminated another 118 studies that were not eligible because they were not about PCC (43), the number of participating women was not stated to ascertain if at least 50% were women (28), or because the condition (24), publication type (12), participants (6) or setting (5) were not eligible. Ultimately, we included 39 studies in this review (Fig. 1). Additional file 2: Table S2 includes data extracted from included studies.

### Study characteristics

Studies were published from 2008 to 2018. Among the 39 studies, clinicians were participants in 5 (12.8%). Of the remaining 34 studies involving patients, women were the sole participants in 22 (64.7%), 93.0 and 94.0% of participants in 2 studies [25, 26], and in 10 (29.4%) studies comprised 50.0 to 69.8%. Most studies were conducted in the United States (17, 43.6%). Others were conducted in the United Kingdom, Canada, Belgium, Netherlands, Japan, Australia, Brazil, Chile and South Africa. Most studies involved qualitative interviews with patients or clinicians (18, 46.2%) or surveys (17, 43.6%), and 4 (10.3%) were observational studies. More than half of included studies investigated maternal care (21, 53.0%). Other studies investigated cancer, diabetes, HIV, endometriosis, dementia, distal radius fracture, overactive bladder, and systemic lupus erythematosus. With respect to objectives, most studies (23, 59.0%) explored patient and/or clinician views about PCC and its determinants. The remainder examined whether PCC took place (8, 20.5%), evaluated interventions meant to support PCC (4, 10.3%), solely identified determinants of PCC (3, 7.7%), or developed a scale to measure PCC (1, 2.6%). Of the 39 studies, 17 (43.6%) were qualitative studies, which inductively explored what women perceived as PCC to truly represent what women believed constitutes PCC. Most studies referred to patient-centred care (23, 59.0%). Others referred to woman- or women-centred care (10, 25.6%), person-centred care (3, 7.7%), family-centred care (2, 5.1%) or patient and family-centred care (1, 2.6%).

### PCC description

Of the 39 included studies, 5 (12.8%) [27, 28, 29, 30, 31] did not define or describe PCC; instead, they explored whether patients experienced PCC. Of the 34 (87.2%) studies that defined or described PCC, none addressed all 6 PCC domains (Table 1). One study involving interviews with mothers of premature babies to explore their views on family-centred neonatal care addressed 5 of 6 domains, and 6 (17.6%) studies addressed 4 of 6 domains [43]. More commonly addressed domains were exchanging information (25, 73.5%) and fostering the patient-clinician relationship (22, 64.7%). Fewer studies addressed making decisions (16, 47.1%), enabling patient self-management (15, 44.1%), or responding to emotions (12, 35.3%). Managing uncertainty was addressed in only 1 study (1, 2.9%) [43]. There were too few studies to compare PCC across different medical conditions, or involving clinicians to compare their PCC priorities with those of women. Among the 34 studies that defined or described PCC, the frequency of addressed domains differed between studies involving largely women (22 studies all women, 2 studies at least 93.0% women) and mixed-gender studies (10 studies of 50.0 to 69.8% women), suggesting that women’s PCC preferences may differ from those of men. In studies involving largely women, 78.0% addressed exchanging information (40.0% mixed-gender), 54.2% addressed fostering the relationship (70.0% mixed-gender), 33.3% addressed each of making decisions and enabling self-management (50.0 and 60.0%, respectively, mixed-gender), 29.9% addressed addressing emotions (50.0% mixed-gender), and 4.2% addressed managing uncertainty (0.0% mixed-gender). No studies identified PCC components not already captured by the McCormack framework.

**Table 1:** PCC domains measured or identified as important in included studies

Study (year, country)	Participants (% women)	PCC domains (n,%)						
		Fostering the relationship	Exchanging information	Addressing emotions	Managing uncertainty	Making decisions	Enabling self-management	Domains per study (n)
		• Discuss roles and responsibilities • Honesty and openness • Trust in clinician competence • Express caring • Build rapport	• Explore needs and preferences • Share information • Provide information resources • Assess and facilitate understanding	• Explore and identify emotions • Assess anxiety or depression • Validate emotions • Express empathy or reassurance • Provide help to deal with emotions	• Define uncertainty • Assess uncertainty (cognitive) • Use emotion-focused management strategies (affective) • Use problem-focused management strategies (behavioural)	• Communicate about decision needs, support and process • Prepare for deliberation and decision • Make and implement a choice and action plan • Assess decision quality and reflect on choice	• Learn and assess • Share and advise • Prioritize and plan • Prepare, implement and assist • Arrange and follow-up	
Maternal care								
Wright 2018 [32] Australia	(clinicians)	–	x	–	–	–	–	1
Afulani 2017 [33] United States	100.0	–	x	x	–	–	–	2
Afulani 2017 [34] United States	100.0	–	x	–	–	x	–	2
Hollander 2017 [35] Netherlands	100.0	–	–	–	–	–	–	0
Balbino 2016 [27] Brazil	60.6	x	–	–	–	–	–	1
Binfa 2016 [36] Chile	100.0	x	–	x	–	–	–	2
Borrelli 2016 [37] United Kingdom	100.0	–	x	–	–	x	x	3
Phillippi 2016 [38] United States	100.0	–	x	–	–	–	x	2
Thompson 2016 [39] Netherlands	(clinicians)	–	–	–	–	x	–	1
Farrell 2015 [40] United States	(clinicians)	x	x	–	–	x	–	3
Galle 2015 [41] Belgium	100.0	x	x	–	–	–	x	3
Larson 2015 [42] United States	100.0	x	x	–	–	–	x	3
Finlayson 2014 [43] United Kingdom	100.0	x	x	–	x	x	x	5
Iida 2014 [44] Japan	100.0	x	–	–	–	x	–	2
Larson 2014 [28] United States	100.0	x	x	–	–	–	–	2
Martin 2014 [25] United States	100.0	–	–	–	–	–	–	0
Bergman 2013 [29] United States	100.0	–	x	–	–	–	–	1
Moore 2013 [45] United States	100.0	–	x	–	–	x	–	2
Van Kelst 2013 [46] Belgium	(clinicians)	–	x	–	–	x	x	3
Iida 2012 [47] Japan	100.0	x	x	–	–	x	–	3
Asai 2011 [48] Japan	(clinicians)	x	x	–	–	–	–	2
Cancer								
Long 2016 [49] South Africa	100.0	–	–	–	–	–	–	0
Kuroki 2013 [50] United States	100.0	–	–	–	–	–	–	0
Sewitch 2013 [51] Canada	53.1	x	x	–	–	–	x	3
Sterba 2013 [52] United States	50.0	–	–	–	–	x	x	2
SintNicolas 2012 [53] Netherlands	51.0	x	x	–	–	–	x	3
Diabetes								
Grohmann 2017 [30] Canada	65.2	–	–	–	–	–	–	0
Thomas 2014 [54] United States	59.7	–	–	x	–	x	x	3
Moran 2008 [55] United Kingdom	54.0	x	–	x	–	x	x	4
HIV								
O’Brien 2017 [56] Canada	100.0	–	x	–	–	–	x	2
Sullivan 2015 [57] United States	100.0	x	x	x	–	–	–	3
Gourlay 2014 [58] United Kingdom	100.0	x	–	x	–	x	–	3
Dementia								
Lerner 2014 [59] United States	69.8	x	–	x	–	x	–	3
Zaleta 2010 [60] United States	61.1	x	x	x	–	x	–	4
Endometriosis								
Apers 2017 [61] Belgium	100.0	x	x	x	–	–	x	4
Dancet 2012 [62] Belgium	100.0	x	x	x	–	–	x	4
Bladder								
Hahn 2017 [63] United States	93.0	x	x	–	–	–	–	2
Fracture								
Constand 2014 [64] Canada	68.2	x	x	x	–	–	x	4
Lupus								
Beusterien 2013 [31] United States	94.0	x	x	x	–	x	–	4
Total (n, % of 34)^a^	–	22 (64.7)	25 (73.5)	12 (35.3)	1 (2.9)	16 (47.1)	15 (44.1)	–

^a^ 34 of 39 studies defined or described PCC

### PCC instruments

Eleven (28.2%) studies employed validated instruments to assess PCC. They included: Endometriosis Care Questionnaire [61, 62], Perceptions of Family Centred Care – Parents and Perceptions of Family Centred Care – Staff [27], Patient Expectations and Satisfaction with Prenatal Care Instrument [41], Patient Perception of Patient Centred Care [64], Women Centred Care Pregnancy Questionnaire [44, 40], Patient Assessment of Chronic Illness Care [54], Communication Assessment Tool [50], Wake Forest Trust Scale [50], Global Rating Scale [53], Japanese Measure of Processes of Care for Service Providers, and Japanese Measure of Beliefs about Participation in Family Centred Service [48]. One study validated a new PCC instrument, the Person Centred Maternity Care Tool [34]. However, as noted, instruments varied in the domains they assessed, and none addressed all 6 McCormack PCC domains [7], suggesting that current instruments may not be fully or accurately assessing PCC. No instruments included PCC components not already captured by the McCormack framework.

### PCC impact

Of the 39 included studies, 11 (28.2%) reported outcomes associated with PCC. These were captured via validated instruments, qualitative interviews, or non-validated surveys created specifically for the study, and were captured either following a PCC intervention or regular consultation. Patient ratings of PCC were found to be positively correlated with health-related quality of life [61], care satisfaction [44, 47, 50], disease understanding [54], sense of control during birth [44, 47], and clinical outcomes including pain reduction [64], functional recovery [64], and physiological child birth [33]. Patient ratings of PCC were negatively correlated with perceived consequences of illness [54]. PCC behaviours displayed by clinicians were positively correlated with patient knowledge [58], patient well-being [58], treatment satisfaction [31], emotional health [31], and use of prevention of mother-to-child transmission services [54]. Additionally, PCC ratings influenced care preferences regarding induction of labour (direction unspecified) [45].

### PCC determinants

Fifteen (38.5%) studies identified challenges or barriers to PCC. Clinician traits such as being present during procedures [37, 46], less than 5 years of experience [40], racism [29, 57], training [58], overconfidence [28], and low self-efficacy [48] impeded the use or effectiveness of PCC. Patient barriers to PCC included language (i.e. whether clinician spoke Spanish) [29], social determinants of health [56], HIV stigma [56, 58], lack of preparation for meetings [36], and discomfort with asking questions [36]. Power struggles within the patient-provider relationship [43] and lack of tools to support patient-provider interaction [25, 46] also challenged PCC.

### Strategies to support PCCW

Four studies examined programs or interventions to support PCCW. Two of these were implementation of PCC models during maternity care: the Patient and Family Centred Care Model [27], which involved an organizational shift towards family being accepted as the primary source of support and strength for newborns, including concepts such as unrestricted access to newborns, service flexibility, cooperation, respect, and increased autonomy for the families; and the Model of Integrated and Humanised Midwifery Health Services, which involved specific activities such as strengthening the patient-clinician relationship, continuous emotional support, encouraging variation in birthing position and pain relief methods, and promotion of mother and child bonding [27]. Other programs were the Nurse Patient Navigation Program for HIV care, aiming to retain women of colour in HIV care by supporting autonomy via orientation, care coordination and regular phone calls from a registered nurse for up to 8 months [57]; and specialized diabetes education sessions with a registered nurse and a dietician that included individualized information about self-care, lifestyle habits, treatment priorities and action plans [30]. Three of these studies reported positive results including improvement in patient perceptions of PCC [27, 30, 57], happiness and connection with clinician [57], and increased self-efficacy for self-management [30]. In contrast, 1 study found that women were unhappy with their care, and there were multiple discrepancies between women’s PCC needs and clinician practices despite the implementation of this program [36].

## Discussion

The aim of this review was to examine how PCCW has been conceptualized or operationalized in prior research. Among 39 studies published from 2008 to 2018 comprised largely of women, most explored what constitutes or influences “patient-centred care”. While some studies employed validated instruments, which varied in how they assessed PCC, no studies thoroughly described PCC based on the 6-domain McCormack framework [7], and none identified PCC components not already identified by that framework. Compared with mixed-gender studies, those comprised largely of women more frequently prioritized exchanging information above other domains, and less frequently prioritized other domains. Studies were too few to compare PCCW across conditions, or to compare patient views about PCCW with those of clinicians. Numerous patient and clinician characteristics impose barriers on PCCW. Few studies tested strategies to support PCCW or evaluated the impact of PCCW; those that did largely demonstrated beneficial impact on patient knowledge, satisfaction, well-being, self-care and clinical outcomes. Thus, it is important to achieve PCCW, but due to a paucity of research, we lack insight on how.

These findings are consistent with a similar review investigating PCCW across 3 conditions (cardiovascular disease, mental health, and reproductive health), where we identified a paucity of primary research on PCCW, and found that exchanging information was the most-addressed domain compared with other domains [23]. These findings are also consistent with research in Germany, where after multiple rounds of surveys with international experts, patient information was rated as the most important PCC dimension [65]. However, the finding that women prioritized exchanging information more than other domains, which contrasted with PCC priorities in mixed-gender studies, is unique from prior research. For example, Binfa et al. reported that women felt uninvolved in decision-making and wanted to ask questions but thought that might upset clinicians [36]. Similarly, Martin et al. found that women expected psychosocial support and wanted clinicians to provide reassurance about postpartum symptoms [25]. These discrepancies underscore that what constitutes PCCW remains unclear, and further research is needed to identify which elements are common, and which need to be tailored for women with different conditions.

Several implications emerge for policy and practice. First, it is surprising that despite demonstrated disparities in women’s health [10–13], advocacy to improve women’s health [14–16], and insight on what constitutes PCC [6, 7], little research has examined PCCW, as was found in our previous review [23]. Expert consensus in the United States [17] and Canada [18] recommended the need for policies that espouse women’s health care needs and priorities. Thus, further research may be needed to examine the content of legislation and policies for women’s health strategies and incentives, information that could prompt policy-makers to better address PCCW in system-level strategies. Second, it is well-recognized that women are under-represented as research participants, which limits the applicability of findings [26]. In addition to analysis of government policies, analysis of research funding agency policies may also reveal if resources are equitably allocated to the study of PCCW. Third, while 11 studies employed validated scales of PCC-related constructs, none addressed all 6 McCormack PCC domains [7]. This raises questions of whether currently available tools are accurately or thoroughly measuring PCC, and potentially limits the ability of health care professionals or organizations to improve PCC if it is not being fully assessed. Further research is needed to generate measures or instruments to evaluate PCC. This research found that PCC priorities may indeed differ between men and women, but due to few included studies, could not determine if PCCW differs across conditions.

This review features strengths and limitations. We employed a review approach most relevant to study goals [19–21], and rigorous review methods that complied with reporting standards [22, 24]. Data were independently reviewed by multiple researchers and the research team to enhance rigour, and reliability of the findings. None disagreed with findings, but helped to enhance clarity in reporting the findings. A few issues may limit the interpretation and use of the findings. We did not search grey literature as that is not typical of a rapid review [20, 21], and due to the methodological challenges that have been identified by others [66, 67]. While our search strategy was comprehensive, we may not have identified all relevant literature because we excluded non-English language studies, and because relevant research may not have been labelled as “patient-centred” or similar terms. We based our assessment on the 6-domain, 31 sub-domain McCormack PCC framework [7], which is not a gold standard, but was rigorously developed, and appeared more comprehensive than other frameworks [6], and provided a reference point for assessing PCC in women’s health research. This PCC framework proved to be relevant because no studies or instruments used in studies to measure PCC identified PCC components not already included in the framework.

## Conclusions

This theoretical rapid review found that, despite worldwide disparities in women’s health, advocacy to improve women’s health and emphasis on PCC, little research has established what constitutes PCCW, or how to implement or measure PCCW. Our analysis suggests that women’s PCC preferences may differ from those of men, but further research is needed to confirm this, and whether PCCW differs across conditions, knowledge needed to inform policies, guidelines and measures aimed at improving health care and associated outcomes for women.

## Supplementary information

**Additional file 1: Table S1.** MEDLINE search strategy.

**Additional file 2: Table S2.** Data extracted from included studies.

## Data Availability

Not applicable.

## Abbreviations

PCC: Patient-Centred Care
PCCW: Patient-Centred Care for Women
PICOT: Participants, Issue, Comparisons, Outcomes, Type of publication
PRISMA: Preferred Reporting Items for Systematic Reviews and Meta-Analyses
