# Supplementary material for: What constitutes patient-centred care for women: a theoretical rapid review
Source: Int J Equity Health. 2019 Nov 26;18:182. doi: 10.1186/s12939-019-1048-5 (PMC6880419; doi:10.1186/s12939-019-1048-5)
Supplement: Supplementary file 1 — Additional file 1: Table S1. MEDLINE search strategy. [file 12939_2019_1048_MOESM1_ESM.docx]

S1 Table. MEDLINE search strategy

1     women's health/ (25422)
2     women/ (14247)
3     female/ (7835541)
4     1 or 2 or 3 (7839777)
5     Patient-Centered Care/ (15651)
6     (patient centered or patient-centered or patient centred or patient-centred).mp. (27001)
7     (person centered or person-centered or person centred or person-centred).mp. (3883)
8     (wom#n centered or wom#n-centered or wom#n centred or wom#n-centred).mp. (450)
9     5 or 6 or 7 or 8 (30272)
10     4 and 9 (8723)
11     limit 10 to (english language and yr="2008 -Current" and "all adult (19 plus years)") (5055)
12     limit 11 to (comment or editorial or interview or lectures or letter or news) (26)
13     11 not 12 (5029)
14     depression/ (99502)
15     13 and 14 (161)
16     cardiac rehabilitation/ (1535)
17     13 and 16 (60)
18     family planning services/ or reproductive health services/ (25063)
19     13 and 18 (28)
20     Preventive Health Services/ (12323)
21     Health Promotion/ (65178)
22     Healthy Lifestyle/ (499)
23     20 or 21 or 22 (76434)
24     13 and 23 (116)
25     14:24/or (325594)
26     13 not 25 (4669)

Note: Results from search line 14 to 24 were used in another review that focused on cardiac rehabilitation, depression, family planning and health promotion, topics that were excluded from this review
