# Supplementary material for: What constitutes patient-centred care for women: a theoretical rapid review
Source: Int J Equity Health. 2019 Nov 26;18:182. doi: 10.1186/s12939-019-1048-5 (PMC6880419; doi:10.1186/s12939-019-1048-5)
Supplement: Supplementary file 2 — Additional file 2: Table S2. Data extracted from included studies. [file 12939_2019_1048_MOESM2_ESM.docx]

S2 Table. Data extracted from included studies

**Maternal Care**

| Study | Research Design | Objective | PCC Term | PCC Definition or Measurement | McCormack PCC elements described or recommended | Findings |
| --- | --- | --- | --- | --- | --- | --- |
| Wright 2018^25^ Australia | Observation of 16 midwives (aged 30-50) in an antenatal clinic | Observe how the principles of woman-centred care are applied in antenatal care | Woman-centred care | Given the observational methods, no strict definition of PCC was used. Analysis was guided by the works of Stapelton (2002a, 2002b) as focusing on communication:   - Who initiated conversations - Who spoke the most - Where conversations took place | **Emotions:** Hearing the woman's voice (exploring worry)  **Information:** Patterns and routines of consultation (greeting, pre-assessment, assessment, post-assessment) and Hearing the woman's voice (setting the agenda)  **Relationship:** Hearing the woman's voice (connecting with the woman,)  **Uncertainty:** Hearing the woman's voice (exploring worry) | Key areas where varying midwife behaviours affected the next step in consultation outcome of care were:   - Patterns and routines of consultation - Language and words used - Hearing the woman's voice |
| Afulani 2017^26^ United States | Development and validation of a tool for measuring PCC among 1387 women (mean age 25.2) | Develop and validate a scale for measuring women's perceptions of patient-centred maternity care | Person-centered maternity care | Defined based on the WHO quality of care framework for maternal and newborn health as care that is:   - Holistic - Responsive - Respectful - Dignified   And emphasizes:   - Experience of care - Communication - Emotional support | **Information, Relationship, and Self-management** were identified by the authors as domains of patient-centred maternity care via literature review to inform survey development | A 30-item scale for measurement of women's perceptions of person centered maternity care was developed and found to have high validity and reliability |
| Afulani 2017^27^ United States | Qualitative focus groups with 58 women (mean age 26) | Identify aspects of care that contribute to a positive or negative child-birthing experience | Person-centered maternity care | Defined based on the Institute of Medicine (US) as respecting and responding to childbearing families' needs, preferences, and values | **Information:** Effective communication identified as influential to women’s perceptions of their care | Four themes were identified as influential to women's perceptions of their care:   - Responsiveness - Supportive care - Dignified care - Effective communication |
| Hollander 2017^28^ Netherlands | Analysis of survey data from 2192 women aged 18+ (mean age 33.1) | Evaluate women’s attributions to caregivers and themselves regarding traumatic child birth | Woman-centred care | **---** | Domains women felt clinicians should have done differently (Percent women who chose that answer):  **Emotions:** "Support me (more/better, emotionally/practically" (29.8%)  **Information:** “Communicate/explain” (39.1%). "Listen to me (more)" (36.1%)  Participants felt that they could have done:  **Decision-making:** "Ask for certain actions/interventions" (26.9%) and  "Refuse certain actions/interventions" (16.5%) | 37% of participants felt that they could have done "Nothing" |
| Balbino 2016^29^ Brazil | Analysis of survey data from 66 parents of newborns (60.6% women, aged 16-50) and 57 professional staff (91.2% women, aged 25-55) | Assess the effects of the patient and family centered care model on parents and clinicians | Patient and family centred | Measured based on 3 domains:   - Respect: family rights in the hospital - Collaboration: partnership of care between parents and clinicians - Support: the way clinician teams offer support to the family | **Information**: Patients increased in this factor post-intervention: “I feel overwhelmed by the information given to me about my child.”  **Relationship:** Clinicians increased in understanding attitude towards the parents’ experiences | Parents significantly increased in their responses regarding all three domains post intervention. These responses mainly indicated an increase in PCC except for an increase in the following question: “I feel like a visitor (rather than a parent) when I come to the hospital |
| Binfa 2016^30^ Chile | Qualitative interviews with 27 postpartum women (aged 16-37), 40 midwives (aged 24-62), and 29 obstetricians (aged 27-68) and analysis of survey data from 1729 postpartum women (mean age 24) | Explore perceptions of professionals and consumers regarding humanised assistance during labour and childbirth | Woman-centred | Defined based on the work of Stapleton and colleagues (2013) and the Chilean Ministry of Public Health guideline and models for care as:   - Safe - Personalised - Humane - Strengthening patient-clinician relationship - Emotional support - Promotion of child-mother bonding - Improving several clinical outcomes - Access to care | **Emotions:** Women felt that clinicians didn’t acknowledge their emotions (ex. Fear)  **Information:** Women felt like they weren’t being listened to. Felt that clinicians may get upset if they ask questions (uncomfortable)  **Decision-making:** Women felt uninvolved in decision-making  **Relationship:** Women felt clinicians had a lack of empathy | Midwives felt that patients were:   - Undereducated regarding childbirth - Not prepared to participate in care |
| Borrelli 2016^31^ United Kingdom | Pre- and post-birth semi-structured interviews with 14 and 12 women (aged 19-43) | Understand first time mothers' expectations and experiences of childbirth care and conceptualize the perspectives of women regarding what makes a good midwife | Woman-centred care | Reference to National Service Framework for Children, Young People and Maternity Services guidelines which address PCC as:   - Meeting the needs of parents, children and their families - Ensuring patients are involved in decision making - Giving patients options about how and where they can give birth | **Emotions:** Addressing uncertainties and insecurities about the labour process and any emotional or physical reactions  **Information:** Accurate and timely information to promote active support and reassurance  **Relationship:** Women expressed a need to trust that clinician is focused on her as an individual, is competent and conveys respect. Establishing a trusting relationship so the patient feels listened to, understood and supported. Ex. Sharing personal stories or notifying a patient when switching shifts to establish respect and trust  Knowledgeable doing: Competent, confident and safe care  **Uncertainties:** Addressing uncertainties and insecurities about the labour process and any emotional or physical reactions | Four themes of a good midwife emerged:   - Promoting individuality - Supporting embodied limbo - Helping to go with the flow: Adapt expectations to the actual birth experience - Providing information and guidance   Characteristics of an ideal midwife were:   - Physical presence - Immediately available presence - Relationship-mediated being - Knowledgeable doing |
| Phillippi 2016^32^ United States | Semi-structured interviews with 50 pregnant women (aged 18 – 35+) | Explore women's perspectives on facilitators of quality of prenatal care | Patient-centred care | Measured via questions about:   - Quality of care - Preference between seeing a midwife versus doctor - Importance of seeing the same midwife each time | **Information:** The second largest theme emerging from women’s’ responses was communication including: “Relaxed atmosphere conducive to communication” “Having time to ask questions without being judged as uneducated” and “Provided information to assist them in making an informed decision”  **Decisions: “**Provided information to assist them in making an informed decision” and “Connection paired with communication enabled informed shared decision making” mentioned as part of communication theme  **Relationship:** The largest theme emerging was connection including: “Treated as a unique and special person” “Connect with clinician to get the care they need/want” “Clinician compatible with needs, culture and beliefs” and “Clinician competency” | **---** |
| Thompson 2016^33^ Netherlands | Semi-structured focus groups with 37 midwives aged 26-62 | Investigate the attitudes and motives of midwives regarding promotion of physiological birth | Woman-centred | Defined as supporting women to trust in the physiological process of birth, and enabling them to make decisions about their birth | Midwives described women centeredness as:  **Emotions:** Having the aim of empowering women to feel confident  **Decisions:** Promoting shared decision making and ensuring women are aware of all their choices  **Relationship:** Establishing trust between them and the woman | **---** |
| Farrell 2015^34^ United States | Analysis of survey data from 216 healthcare providers (69.8% women, aged 29-73) | Compare the practices of clinicians during clinical counselling for first trimester screening with the needs and preferences of patients | Patient-centred | Measured using open ended questions about:   - Medical knowledge - Informed consent and patient education - Communication skills specific to FTS - Perception of patients decision making needs and priorities regarding FTS | A statistically significant discrepancy was present between the preferences of patients and clinicians regarding:  **Emotions:** Anxiety and stress associated with FTS (clinicians viewed these as more important than they were to women)  **Information:** Risks associated with invasive diagnostic testing. Information about resources to raise child with Down Syndrome  **Decision-making:** Exploring personal beliefs about testing choices. Options to continue or end the pregnancy if fetal aneuploidy was subsequently confirmed. The only factor that clinicians and patients agreed on was the need to consider patients’ personal values and beliefs about raising a child with Down Syndrome prior to undergoing FTS  **Self-management:** Understanding follow-up testing options if FTS indicated increased aneuploidy risk.  **Uncertainties:** Knowledge about the chance and meaning of a false positive result. | Clinicians described experience as a determinant for PCC.  The only factor that clinicians and patients agreed on was the need to consider patients personal values and beliefs about raising a child with Down Syndrome prior to undergoing FTS |
| Galle 2015^35^ Belgium | Analysis of survey data from 139 pregnant women from vulnerable populations (aged 18-40) | Assess the expectations and satisfaction of vulnerable pregnant women with antenatal care | Women-centred care | Measured based on 2 domains:   - Expectations: complete, continued, personalized care, availability of other services - Satisfaction: information, provider care, staff interest, and system characteristics | **Information:** Women were less satisfied with “information” and indicated a lack of communication  **Relationship:** Women were satisfied with “staff interest” “provider care” and the clinical aspects of their care | Women’s satisfaction with patient-clinician relationship:   - "staff interest" = "provider care" > "information" - Lack of communication   Satisfied with clinical aspects of care |
| Larson 2015^36^ United States | Analysis of data collected using a discrete choice experiment with 3003 postpartum women (mean age 27.1) | Assess the preferences of patients regarding delivery care and examine what contributes to their variation, particularly those factors that can be changed | Patient-centred | Defined as:   - Responsive to individual preferences, needs and values, - Effective communication to reduce information asymmetry - Promotion of care uptake and adherence   Measured via DCE attributes:   - Clinicians attitude - Medical knowledge - Privacy during delivery | **Relationship:** “Medical knowledge” | Women’s valuation of attributes:  Clinician kindness > Medical knowledge > facility equipment and drugs, medical knowledge, facility is clean and tidy, privacy for delivery, cost |
| Finlayson 2014^37^ United Kingdom | Interviews with 12 mothers of premature babies (aged 21 – 40) | Explore the perceptions of mothers on family centred care in neonatal intensive care units | Family-centred care | Measured based on Harrison's "Principles of Family Centered Neonatal Care” providing key PCC strategies for parents and clinicians to work together towards:   - Open and honest communication - Parents to make informed choices based on access to the same facts and uncertainties as those available to clinicians - In high morbidity / mortality / medical controversy situations, parents make decisions regarding aggressive treatment - Information about adverse pregnancy outcomes provided and treatment preferences considered if premature delivery - Acknowledge and alleviate the pain of infants - Appropriate environment is maintained - Safety and efficacy of medical treatments - Promote parenting skills - Promote long term follow up for survivors - Treatment  based on compassion. Both over- and under-treatment may harm the infant | **Emotions:** Consistent powerless, uninformed and silent observation of the baby, machinery, and staff (anxious surveillance theme)  **Information:** Daily turnover of staff resulted in varying care strategies and left women feeling uninformed and out of touch with their child’s care (consistently inconsistent)  **Decisions:** Women didn't take part in decision making because they felt they were in no place to and left it to the experts (difference to the experts)  **Relationship:** Initial deference to clinicians resulted in a continuous submissive relationship where patients felt an imbalance of power. (power struggles) Difficulty having open and honest conversations with clinicians due to fear of appearing overly emotional or unstable leading to consequences regarding the babies care, especially regarding consent for care ex. Baby’s first bath (muted relations)  **Uncertainties:** Postpartum women didn’t feel like mothers because they weren’t directly caring for newborns and weren’t able to take them home | A global theme of “finding my place” was identified, along with subthemes:   - Mothering in limbo - Consistently inconsistent - Power struggles - Anxious surveillance - Muted relations - Difference to the experts |
| Iida 2014^38^ Japan | Comparison of survey data from 280 women receiving antenatal care led by a midwife (n=149, mean age 33.2) versus by an Obstetrician (n=131, mean age 31.7) | Investigate women’s perceptions of WCC, care satisfaction, sense of control, and attachment to newborns in different birthing settings | Women-centred care | Defined based on the Horiuchi model (2009) as a woman-caregiver relationship which influences a woman's birth experience with a goal of achieving her wellbeing and empowerment, and based on subscales: Respect, Encouragement, Effective interaction, Shared decision-making, Non-threatening manner and Trusting the caregiver | **Emotions:** ↓ "I felt fearful" highly correlated with ↑ WCC perception  **Information:** Communication patterns at antenatal check-up and "talked the same amount as the caregiver" were determinants of WCC  **Relationship:** ↑ "no feelings of powerlessness" associated with ↑ WCC perception. Feelings of power were a determinant of WCC  **Self-management:** Communication patterns at antenatal check-up were a determinant of WCC | ↑ WCC perception was highly correlated with:   - ↑ Care Satisfaction - ↑ Sense of control - ↑ Infant attachment - ↑ "no feelings of powerlessness" - ↓"I felt fearful"   Determinants of WCC perceptions were:   - Birthing facility - Communication patterns at antenatal check up - "talked the same amount as the caregiver" - Feelings of power |
| Larson 2014^39^ United States | Analysis of survey data from 855 postpartum women (mean age 27.66) | Explore women's perceptions of quality of care during their last obstetric visit and the impact of their expectations and experiences | Patient-centred care | Assess based on the questions regarding patients experience with:   - Being greeted and spoken to respectfully - Knowledge and competence of clinicians - Clarity of explanations - Privacy | Women rated quality of care factors (% women rating each quality as excellent/very good):  **Information:** “Clarity of explanation” (32.86%)  **Relationship:** “Knowledge and competence of clinicians” (35.50%) | Quality of care ratings (Percent women rating each quality as excellent/very good):   - Average: 18.65 - 116 women rated as excellent - Greeted and talked to respectfully (37.93%) - Knowledge and competence of clinicians (35.50%) - Clarity of explanation (32.86%) - Privacy (36.79%)   Determinants of quality ratings investigated:   - Clinician confidence (↓ Quality of care) - Disrespectful treatment or abuse (↓ Quality of care) - Complication during birth (no effect) |
| Martin 2014^40^ United States | Focus group interviews of 45 postpartum women (mean age 31) and 13 obstetric clinicians (mean age 32) | Explore women’s experiences with postpartum care and investigate factors that enhance postpartum preparation and maternal outcome | Patient-centered | **---** | **Emotions:** Women expected psychosocial support. Clinicians felt they didn’t have the training or time to provide psychosocial support  **Information:** Women wished clinician warned them of postpartum symptoms to provide reassurance that they were normal. Clinicians felt disclosing normal consequences of birth which would resolve themselves is a waste of time and that more information should be given during prenatal period. Clinicians and patients felt that a postpartum informational tool should be provided  **Relationship:** Women felt that follow-up doctor didn’t understand what they went through (change from delivery doctor). Clinicians felt that women didn’t feel comfortable opening up to recovery doctor because they had never met before  **Self-Management:** Clinicians felt that Women had inaccurate expectations of recovery | Four major themes were extracted from the FGDs:   - Lack of postpartum period knowledge - Lack of continuity of care - Disconnect between providers and postpartum mothers - Bridging the gap/suggestions for improvement |
| Bergman 2013^41^ United States | Semi-structured interviews with 48 prenatal or postnatal women | Examine PCC dimensions relating to patient-provider communication | Patient-centred care | Acknowledged the lack of an invariable definition of PCC. Focused on patient-provider communication as the main relevant domain to the Hispanic population | **Information:** Patients viewed “ensuring information is understood” as an aspect of PCC  **Relationship:** Patients valued effective medical care and a friendly relationship (Ex. Receiving patients well, smiling, eye contact, introductions, not rushing). This was rated most important aspect by 42% of patients | Five themes emerged when patients were asked what PCC means to them:   - A friendly relationship - Effective medical care - Spanish language spoken - Ensuring information is understood - Elimination of racism |
| Moore 2013^42^ United States | Pre- and post-induction interviews with 29 women aged 21-41 | Understand women's perspectives on induction of labour (IOL) and factors influencing their decisions, knowledge about risks and benefits and postpartum experience | Patient-centred | Defined based on Patient-Centered Outcomes Research Institute and U.S. Agency for Healthcare Research and Quality. Pre-induction measurement questions focused on decision making whereas post-induction interview questions reflected on the birth experience:   - Advise to future patients - Advise to clinicians - Three key “take-away” messages | **Emotions**: Relief from discomfort/anxiety identified as a pre-induction theme  **Information:** Women suggested that Information about IOL should be provided at childbirth education classes and that Future patients should seek information well in advance of making a decision  **Decisions:** Lack of informed decision making identified as a pre and post induction interview theme. Women suggested that Future patients should seek information well in advance of making a decision and Future patients shouldn’t just say yes because they think there is no choice  **Relationship:** Trust in clinician identified as a pre and post induction interview theme  **Uncertainties:** Diminished potential or actual risks identified as a pre-induction theme | Five major themes were extracted from both pre and post induction interviews.  Non PCC Pre-induction themes:   - Safety of baby   Non-PCC Post induction themes:   - IOL as part of a checklist - Satisfaction with IOL decision |
| Van Kelst 2013^43^ Belgium | In-depth interviews with 12 midwives aged 25-56 | Investigate views of midwives regarding actual versus ideal maternity care | Woman-centred care | Described as care that the mother herself desires with her individual needs as the focus:   - Not having unnecessary interventions - Making informed choices - Receiving continuity of care | **Decisions:** Women-centred care described as involving a focus on women’s needs including no unnecessary interventions, informed choice (i.e. making sure women had enough information during prenatal care or even pre-contraception), Midwife and obstetrician working together as equal partners, and  Intra-collegial harmony | Midwives responses revealed 5 themes of ideal maternity care:   - Woman-centred care - Cultural change - Change in culture of the workplace needed to facilitate PCC - Support: Midwives reported needing support via education and staffing improvements to facilitate PCC - Midwife and obstetrician working together as equal partners - Intra-collegial harmony |
| Iida 2012^44^ Japan | Analysis of survey data from 481 postpartum women (mean age 31.2) | Investigate women’s perceptions of WCC, care satisfaction, sense of control, and attachment to newborns | Women-centred care | Defined based on the Horiuchi model (2009) as a woman-caregiver relationship which influences a woman's birth experience with a goal of achieving her wellbeing and empowerment, and including 4 elements:  Respect, Safety, Holism, and Partnership | **Decision-making:** ↑ **“**help in decision making” rating associated with ↑ care satisfaction and control  **Relationship:** ↑ “Feelings of encouragement” “Effective interaction” and “Trusting the caregiver” associated with ↑ care satisfaction and control | Overall women had high WCC-questionnaire scores and perceived that they were receiving WCC |
| Asai 2011^45^ Japan | Analysis of survey data from 710 nurses in the NICU (98.5% women, mean age 30.3) | Identify predictors of nurses' family centred care practices | Family-centred care | Defined based on the Institute for Family centered Care (1992) as including 4 core concepts:   - Dignity - Respect - Information sharing - Participation - Collaboration | **Information:** Nurses ranked information needs lower than relationship needs in the following order: "communicating specific information about the child" > "providing general information."  **Relationship:** Nurses ranked "showing interpersonal sensitivity" as more important than information needs, but less important than dignity and respect | Rankings were as follows:  From the Japanese Measure of Processes of Care for Service Providers:  "Treating people respectfully" > "showing interpersonal sensitivity" > "communicating specific information about the child" > "providing general information."  From the Measure of Beliefs about Participation in Family Centred Service: "family centred care principles" > "benefits of FCC” > “practical feasibility of implementing FCC” > "absence of negative outcomes" > "self efficacy"  The most significant predictors of FCC were: self-efficacy, experience, PCC principles and 24 hour parental visiting policy |

**Cancer**

| Study | Research Design | Objective | PCC Term | PCC Definition or Measurement | McCormack PCC elements described or recommended | Findings |
| --- | --- | --- | --- | --- | --- | --- |
| Long 2016^46^ South Africa | Semi-structured interviews with 28 women with cervical cancer aged 30 – 73 | Identify informational needs of women undergoing brachytherapy to inform PCC guidelines | Patient-centred care | **---** | **Emotions:** Some patients felt scared to read treatment related information  **Information:**  Some patients wanted more information.  Patients suggested holding information sessions prior to treatment  **Decisions:** Patients wanted to be given an opportunity to ask questions before signing consent, feel informed and comfortable to ask, and realize the decision is shared | Four themes identified:   - Informational needs - Disposition towards treatment - Psychological experience - Physical experience |
| Kuroki 2013^47^ United States | Analysis of survey data from 100 women with gynecologic cancer (cervical, endometrial, fallopian tube, ovarian, peritoneal, vaginal or vulvar cancer; mean age 58.2) using the Communication Assessment Tool and Wake Forest Trust Scale Women | Characterize patients’ experiences of gynecologic cancer diagnosis disclosure | Patient-centred | **---** | **Emotions:** Trust in clinician  **Information: “**Conversation pace set by patient” “Question period” “Clinician considered patients’ prior knowledge” Consideration of patient needs during disclosure. “Flow of conversation dictated by patients’ reaction” “Clinician understood patients’ main health concerns”  “Use of body language and non-verbal cues to indicate bad news was forthcoming”  **Relationship: “**Trust in clinician”  **Self-management:** “Follow-up” | Factors that improved patient satisfaction ratings are listed within domains  Mean score = 86.5/100 |
| Sewitch 2013^48^ Canada | Focus group discussions with 66 colonoscopy patients aged 22 - 82 (50% women). Results were used to create quality indicators which were then compared to a standardized measurement of PCC tested on 402 colonoscopy patients (mean age 59.7, 56.2% women) | Identify quality indicators of PCC and their importance ratings based on patients’ experiences and perceptions of colonoscopy care and compare these findings with the Global Rating Scale (informed by staff opinions) | Patient-centred care | Measured via interviews informed by a blueprint of topics including interactions with care givers, opportunities to ask questions, discussion during procedure, explanations, technical skills, comfort and convenience, and receiving information (including about follow up appointments) | **Emotions:** Comfort was crucial for easing anxiety  **Information:** Communication identified as a major theme  **Self-management:** Patients wanted follow-up care especially if complications presented | Three themes were identified:   - Communication - Comfort - Service environment |
| Sterba 2013^49^ United States | Semi-structured interviews with 26 patients with colorectal cancer aged 43 – 82 (50% women) | Explore patients’ experiences of colorectal cancer care | Patient-centred care | Measured via semi-structured interview prompts relating to:   - Diagnosis - Treatment planning process - Expectations and care/treatment   Participants were asked to describe their experiences with care since they have been diagnosed | **Emotions:** Patients expressed confusion about diagnosis and reported that clinicians gave them hope when they were being pessimistic  **Information:** Communication during diagnosis was brief, frank and matter-of-fact.  Communication during treatment focused on symptoms when with the participant and focus on psychosocial wellbeing when with family  **Decisions:** Patients thought that chemotherapy was the only option  **Self-management:** Gained control over their course of treatment  **Uncertainties:** Took comfort in odds ratios because beating the odds > uncertainty. Perceived support as positive when hopeful and negative when uncertain | **---** |
| Sint Nicolaas 2012^50^ Netherlands | Analysis of pre- and post- colonoscopy Global Rating Scale survey data from 1509 patients aged 52 – 63 (51% women) | Evaluate patients’ colposcopy experiences and factors that affect care satisfaction | Patient-centred care | Measured using questions from 5 categories:   - Overall satisfaction - Accessibility and timeliness - Interpersonal skills - Comfort and privacy - Information and aftercare | **Information:** 88% reported being given an opportunity to ask questions before the procedure. 75% were satisfied with verbal communication of results post procedure  **Relationship:** 92% were satisfied with the manner of the endoscopist  **Self-management:** Patients felt informed about what to do if problems occurred after discharge | Patients were overall satisfied with colonoscopy and would return for next treatment |

**Diabetes**

| Study | Research Design | Objective | PCC Term | PCC Definition or Measurement | McCormack PCC elements described or recommended | Findings |
| --- | --- | --- | --- | --- | --- | --- |
| Grohmann 2017^51^ Canada | Specialized diabetes education sessions were conducted between nurses, dieticians and 23 patients with diabetes over the age of 40 (65% women). Interviews were conducted on an individual basis one year into the education session intervention | Explore patients' health care experiences with diabetes education teams integrated into primary care settings | Person-centred care | **---** | **Emotions:** Considerate of patients’ fears  **Information:** Listening, responding to specific questions, providing education and focusing on learning needs  **Decisions:** Shared decision making: partnership between educators  **Relationship:** Patient-provider relationship: Intangible treatment by care providers, considerate of patients’ time, choices, fears, opinions, eating patterns, non-judgemental and helpful  **Self-management:** Increased awareness, skills, and knowledge were created or gained through relationships of patients with diabetes educators. Participants described increased confidence and self-efficacy in management, medications and self-care | Other themes identified were:   - Preference for one on one care rather than group classes |
| Thomas 2014^52^ United States | Analysis of survey data from 87 patients aged 24 – 98 (59.7% women) using Patient Assessment of Chronic Illness Care (PACIC). Survey results were used to capture patient centeredness via calculation of "gap" scores | Evaluate patients' perceptions of chronic care quality, measure PCC, and assess associations between PCC and illness representations | Patient-centred | Measured via PACIC-5A where patient preferences for provider support is measured based on the 5 A's:   - Assess: patient behaviour - Advise: patient counselling - Agree: agreement between patient and provider about realistic goals - Assist: assisting the patient during his/her lifestyle changes - Arrange: arranging follow-ups | **Information:** “Advise” relates to patient counselling. Across all categories, PCC was associated with ↑ disease understanding and ↓ perceived consequences of illness  **Relationship: “**Agree” refers to agreement between patient and provider about realistic goals  **Self-management:** “Assist” implicates assisting the patient during his/her lifestyle changes and  “Arrange” refers to arranging follow-up visits | PCC measurements were representative of consistency between provider frequency of a behaviour and patients' importance rating of that behaviour. The consistency order was: Advise (mean score 0.98 ± 0.97) > Assess > Assist > Agree > Arrange (mean 0.90 ± 0.95) |
| Moran 2008^53^ United Kingdom | Observation of physician consultations with 44 patients with type 1 and 2 diabetes (54% women, aged 19-82) paired with analysis of patient survey data | Assess the use of patient-centred care behaviours in clinicians with no previous training in these techniques, to establish the need for this training | Patient-centred care | Based on the works of Mead (2002) and Epstein (2005):  PCC models of care enable effective communication with patients by attending to psychosocial and physical needs, building a sense of partnership and positive regard, and facilitating their active involvement in decision making  The authors also refer to the 6 components of PCC outlined by Stewart and colleagues 1995 and 2003:   - Exploring the disease and illness experience - Understanding the whole person - Finding common ground regarding management - Incorporating prevention and health promotion - Enhancing the doctor-patient relationship - "being realistic" about personal limitations and issues such as availability of time and resources | **Information:** Satisfaction was correlated with ↓ "patient asking questions" Patient's "assertive responses" were correlated with ↓ "information giving"  The most frequent doctor-patient utterance types were:   - "Additional responses" Ex. facilitative language, statements of agreement etc. - Information giving: correlated with patient “asking questions” - Closed ended questions (21%) - Doctor’s had a higher mean number of utterances than patients (mean doctor:patient ratio 22:17).   **Relationship:** Satisfaction was correlated with ↑ doctor partnership building. Patient's "assertive responses" were correlated with:   - ↑ Doctors' "partnership-building" - ↓ "supportive talk" | Patient-centred talk was one of the least frequent (10%) |

**HIV**

| Study | Research Design | Objective | PCC Term | PCC Definition or Measurement | McCormack PCC elements described or recommended | Findings |
| --- | --- | --- | --- | --- | --- | --- |
| O’Brien 2017^54^ Canada | Qualitative focus groups with 77 women with HIV aged 16+ (67% aged 31-50) | Examine perceptions of women seeking HIV care on women-centred HIV care in Canada | Women-centred HIV care | Based on Hudon (2011), the authors focused on women's care needs as a patient, person, woman, and a woman living with HIV | **Emotions:** Women's needs for basic care competency include empathy. Patient-centred care includes considering mental health  **Information:** Women's needs for basic care competency includes up to date knowledge  **Decisions:** Patient-centred care includes shared decision making  **Relationship:** Patient-centred care includes patient-provider relationships | A conceptual model of women-centered HIV care was created including:   - Women's needs for basic care competency - Patient-centred care - Specific considerations for women living with HIV |
| Sullivan 2015^55^ United States | Semi-structured interviews with 21 African American or black women with HIV aged 27-62 | Explore patients' experiences of care from a nurse guide | Patient-centred care | Measured based on interview prompts about:   - Changes in hope and stigma - Communication with providers - Relationship with the nurse guide - Psychosocial support | Women’s reports regarding the nurse guide included:  **Emotions:** Learned that HIV was not shameful and reduced emotions such as fear and enhanced hope  **Information:** Regarding "communication with providers" women felt that other providers left them feeling confused or lacking information, whereas the nurse provided clarity. Patients reported learning how to effectively ask their doctors questions/talk to them and be more informed  **Relationship:** Found nurse guide’s friendliness and concern to be genuine and made them feel cared for  **Self-management:** Learned how to effectively ask their doctors questions/talk to them and be more informed  **Uncertainties:** For"communication with providers" women felt that other providers left them feeling confused or lacking information, whereas the nurse provided clarity | **---** |
| Gourlay 2014^56^ United Kingdom | In-depth interviews with 21 women aged 20 – 40+ and participatory learning and action group activities with parents in antenatal care including 30 women and 31 men aged 19-59 | Examine the current nature of patient-clinician relationships within the prevention of mother-to-child transmission (PMTCT) of HIV services, identify their influence on service uptake and provide recommendations to optimize patient-provider relations | Patient-centred care | Defined based on Mead and Bower including 5 domains:   - Bio-psychosocial perspective - Patient-as-person - Doctor-as-person: Self-awareness of the doctor - Therapeutic alliance: Personal bond between clinician and patient - Sharing power and responsibility: Shared decision making   This framework also includes influencing factors:   - Doctor factors (personality, gender, age) - Patient factors (attitudes or expectations, age, knowledge) - "shapers" (cultural norms) - Professional context (performance incentives, government policy) - Consultation-level influences (workload pressures, time limitation) | **Emotions:** Psycho-social support mentioned as part of decision making processes  **Information:** Interactions tended to enhance patient knowledge. Decision-making processes involved clear and non-threatening communication by providers and willingness by patients to engage in discussion and clarify information  **Decision-making:** Communication, psycho-social support, power balance, clear and non-threatening communication by providers and willingness by patients to engage in discussion and clarify information to enable shared decision making  **Relationships:** Decision making processes involved power balance. Features of care included therapeutic alliance | Interactions tended to be shaped by structural environment (service type, resources, training, salaries) and led to enhanced patient knowledge, well-being, and use of PMTCT services |

**Dementia**

| Study | Research Design | Objective | PCC Term | PCC Definition or Measurement | McCormack PCC elements described or recommended | Findings |
| --- | --- | --- | --- | --- | --- | --- |
| Lerner 2014^57^ United States | In-depth group education sessions followed by one-on-one follow up sessions between genetic counsellors, physicians, and 262 individuals with first degree relatives affected by Alzheimer’s Dementia (69.8% women, aged 33 - 86) | Examine communication patterns during genetic counselling and determine how teaching, counselling and psycho-educational conceptual models were represented | Patient-centred | Defined based on Mead and Bower including 5 domains:   - Bio-psychosocial perspective - Patient-as-person - Doctor-as-person: Self-awareness of the doctor - Therapeutic alliance: Personal bond between clinician and patient - Sharing power and responsibility: Shared decision making | **Emotions:** Clinician utterances involved 8% psychosocial factors. Patient utterances involved 22% psychosocial factors.  **Information:** Clinician utterances included 56% providing biomedical information and 12% effort in engaging patients. Patient utterances included 5.5% biomedical questions, 20% giving biomedical information and 39% positive statements to clinicians (acknowledgment of what the provider had just said)  **Relationship:** Psychosocial patient centred style involved providers building partnerships with patients | Clinician utterances included (expressed 5.2 times more discrete thoughts than patients).  Three patterns of interaction were identified (frequency):   - Biomedical Provider Teaching (40%): Patients gave significant amounts of biomedical information, made more positive utterances and expressed less emotional thoughts. Communication driven by provider and biomedical focused - Biomedical patient-driven (34.4%): Significant amount of biomedical questions asked, procedural talk and partnership building. Communication driven by patients - Psychosocial patient centred (25.6%) Providers focused on asking psychosocial questions and building partnerships with less of an emphasis on biomedical information. Higher number of statements from participants (high verbal engagement), and patients shared more psychosocial information |
| Zaleta 2010^58^ United States | Analysis of audio recordings of dementia diagnosis with 10 physicians, 54 patients (33 women, mean age 74.13), and 54 companions | Investigate clinicians use of patient-centred approaches to dementia diagnosis, and whether their approaches varied based on patient characteristics | Patient-centred | Defined as exploring patients’ feelings, asking for patient perspectives, enhanced feelings of partnership, a common understanding between patient and clinician, clinician expression of empathy, support, and reassurance, preserving the dignity of patients | **Emotions:** Emotional rapport building involved offering statements of reassurance or optimism, expressing concern or worry, empathy and legitimizing patients’ feelings  **Information:** Positive rapport building involved physician agreement/positive affirmation of patient statements by saying "yes," "absolutely", showing approval towards the patient, and laughing or telling jokes. Facilitation and patient activation involved signalling attention and interest in what the patient is saying without taking over the conversation by saying "mm hmm" or "okay", confirming their own understanding of a statement by asking "do I have that right?”  **Relationship:** Emotional rapport building involved offering statements of reassurance and conveying a sense of partnership with the patient | Frequency of physician PCC behaviour categories (ordered from most to least frequent):   - Positive rapport building - Facilitation and patient activation - Emotional rapport building |

**Endometriosis**

| Study | Research Design | Objective | PCC Term | PCC Definition or Measurement | McCormack PCC elements described or recommended | Findings |
| --- | --- | --- | --- | --- | --- | --- |
| Apers 2017^59^ Belgium | Analysis of survey data from 108 women with endometriosis (mean age 35.4) via Endometriosis Care Questionnaire (Dancet, 2011) and Endometriosis Health Profile-30 (EHP-30) | Examine women's experiences with patient-centred endometriosis care and how they correlate to health related quality of life | Patient-centred care | Measured via 10 dimensions from Endometriosis Care Questionnaire   - Respect for patients' values, preferences and expressed needs - Coordination/integration of care - Information, communication and education - Physical comfort - Emotional support and alleviation of fear and anxiety - Involvement of significant others - Continuity and transition - Access to care - Technical skills - Endometriosis clinic staff | **Emotions:** Patients rated emotional support and alleviation of fear and anxiety the worst (62.1)  **Relationship:** Patients rated endometriosis clinic staff the best (mean score 4.8) | Mean Endometriosis Care Questionnaire score was 38 on a scale of 0 (best score) to 100 (worst score). Worse overall scores were associated with lower levels of health related quality of life |
| Dancet 2012^60^ Belgium | Analysis of survey data from 335 women with endometriosis (mean age 34.3) using the Endometriosis Care Questionnaire (Dancet, 2011) | Assess patient-centred endometriosis care to identify clinic-specific targets for improving PCC | Patient-centred | Measured via 10 dimensions from Endometriosis Care Questionnaire   - Respect for patients' values, preferences and expressed needs - Coordination/integration of care - Information, communication and education - Physical comfort - Emotional support and alleviation of fear and anxiety - Involvement of significant others - Continuity and transition - Access to care - Technical skills - Endometriosis clinic staff | Patients rated the following factors:  **Emotions:** “emotional support and alleviation of fear and anxiety” **Information:** “information communication and education”  **Relationship:**  “technical skills” “endometriosis clinic staff”  **Self-management:**  “continuity and transition” and “coordination and integration of care” | Overall scores did not differ significantly between clinics. Patients’ rankings are as follows: Respect for patients’ values, preferences and needs > technical skills > information, communication and education > endometriosis clinic staff > continuity and transition > access to care > coordination and integration of care > involvement of significant others > physical comfort > emotional support and alleviation of fear and anxiety |

**Other**

| Study | Research Design | Objective | PCC Term | PCC Definition or Measurement | McCormack PCC elements described or recommended | Findings |
| --- | --- | --- | --- | --- | --- | --- |
| Hahn 2017^61^ United States  Overactive Bladder | Observation of consultations between 42 patients (93% women, aged 23 – 85) and 17 physicians | To conduct a sociolinguistic analysis of video recorded patient-clinician interactions | Patient-centred communication vs. Physician-centred communication | Measured from aspects of patient-physician dialogues including the use of ask-tell-ask sequences:   - Ask: An open-ended question with an elaborate patient response - Tell: Physician explains something based on patient response - Ask: Confirmation/recap question   Discussion of quality of life described as a crucial part of PCC behaviour given the nature of overactive bladder. | **Information:** 83% of physician questions were closed ended (patients often gave detailed answers). 40% of visits included discussion of quality of life, 19% were physician initiated  **Relationship:** Dialogues were predominantly physician-centered with physicians speaking 62% of the words  **Self-management:** 40% of visits included discussion of quality of life, 19% were physician initiated.  Physicians who used 2+ successful as-tell-ask sequences were more likely to initiate quality of life discussions | **---** |
| Constand 2014^62^  Canada  Distal Radius Fracture | Analysis of survey data collected via Patient Perception of Patient Centred Care Questionnaire (68.2 % women, mean age 57.11) | Explore patients’ perceptions of PCC, importance ratings of PCC elements, whether perceptions changed over the course of care, and whether they correlated with clinical outcomes | Patient-centred care | Measured using the Patient Perception of Patient Centred Care Questionnaire including questions about whether:   - Clinician is aware of reason for visit - Clinician understood importance of the visit - Main problem was discussed - Patient felt understood - Communication ranking - Satisfaction with discussion of problem - Explanation from doctor about the problem - Patient agrees with clinician’s opinion - Chance to ask questions provided - Clinician asked about goals - Treatment and management explained to patient - Roles were discussed - Patient felt empowerment - Patient felt cared for and that there was a partnership | **Information:** Communication subscale involved doctor understanding the importance of their visit and the reason for their visit  **Decisions:** Patients felt that doctors did not encourage them to participate in their care or take an active role in decision making  **Relationships:** Partnership subscale involved: Patients felt that doctors did not explore their goals for treatment, encourage them to participate in their care, discuss specific clinician or patient roles, or take an active role in decision making  **Self-management:** Patients felt that doctors did not explore their goals for treatment | Patients’ rated Communication > Partnership during baseline  No significant change in score from baseline to follow up.  Small significant correlation between higher perceptions of PCC and clinical outcomes such as pain reduction and functional recovery |
| Beusterien 2013 United States^63^  Systemic Lupus Erythematosus (SLE) | Analysis of survey data from 302 patients with Systemic lupus erythematosus (94% women, mean age 46.3) | To develop a conceptual model of the association between patient-clinician relationship and clinical outcomes based on patient perceptions | Patient-centred care | The survey was designed based on components of PCC identified by patients and clinicians:   - Patient-clinician interactions - Being involved in treatment decision making - Physician bedside manner - Physician helps them to better understand the lupus condition - Satisfaction with physician - Disease control and severity - How hopeful they are about their future health | **Emotions:** Physician interactions led to ↑ emotional health and directly affected health and hope (depression, hopeful about future)  **Self-management:** Physician interactions directly effected treatment and symptom control, and current general health | A conceptual model illustrating the relationship between patient-centred care and patient outcomes was created. Physician interactions lead to ↑ treatment satisfaction |
